# Clozapine and tuberculosis treatment: a case report and literature review

**DOI:** 10.3389/fpsyt.2025.1597895

**Published:** 2025-07-09

**Authors:** Neel Swamy, Jennifer Jepsen, Brian J. Werth, Anna Sunshine, Clayton English

**Affiliations:** ^1^ UW Medicine, Seattle, WA, United States; ^2^ Department of Pharmacy, University of Washington School of Pharmacy, Seattle, WA, United States; ^3^ Department of Psychiatry and Behavioral Sciences, University of Washington School of Medicine, Seattle, WA, United States

**Keywords:** tuberculosis, clozapine, case report, rifampicin, schizophrenia, drug-drug interaction

## Abstract

**Introduction:**

To date, clozapine is the only antipsychotic approved by the United States Food and Drug Administration (FDA) for the management of treatment-resistant schizophrenia. People with serious mental illness are at higher risk of developing tuberculosis and have worse tuberculosis recovery outcomes compared to the general population. First-line regimens for acute tuberculosis often include rifamycins and isoniazid, both of which impact clozapine metabolism and levels through induction or inhibition of the hepatic cytochrome P450 (CYP450) enzyme system. There is limited evidence, mostly from case reports, to guide clinicians in managing clozapine alongside anti-tuberculosis therapy (ATT).

**Literature review:**

We present 5 case reports of patients with schizophrenia or schizoaffective disorder who continued clozapine while receiving ATT. In most of the case reports (*n* = 3), the ATT regimen included both rifampicin, a CYP450 inducer, and isoniazid, a CYP450 inhibitor. We also review pharmacokinetic properties of rifampicin and the potential impact of rifamycin-based regimens on clozapine metabolism and levels.

**Case presentation:**

We present the case of a 35-year-old prescribed clozapine for 4 years prior to being diagnosed with pulmonary tuberculosis. The patient continued clozapine and was closely followed in both the inpatient and outpatient settings while completing a 6-month course of rifampicin, isoniazid, pyrazinamide, and ethambutol. During ATT, the patient had clozapine and norclozapine levels measured at least once monthly and maintained stability in their psychiatric symptoms through adjustment of clozapine and adjunctive antipsychotic dosages.

**Conclusion:**

Our case supports previous reports that ATT can influence clozapine levels. Clozapine dose adjustments will likely be required to maintain clinical stability and prevent adverse effects, but the management appears to be patient-specific. We recommend closely monitoring patients’ clinical status and clozapine levels during and after ATT to optimize outcomes.

## Introduction

Schizophrenia is a chronic mental disorder associated with impaired psychosocial functioning, adverse cardiometabolic outcomes, and reduced life expectancy (1). While schizophrenia affects ~1% of the global population, it is among the ten leading causes of disability, particularly among people between 25 and 54 years (2). Although antipsychotic medications are the cornerstone of treatment for schizophrenia, 1 in 3 people living with schizophrenia will continue to experience symptoms despite trialing multiple, first-line antipsychotics (3).

Clozapine remains the only FDA-approved antipsychotic for the management of treatment-resistant schizophrenia (TRS) (4, 5). Despite metabolic side effects, clozapine significantly lowers all-cause mortality and the risk of recurrent psychiatric hospitalization (6, 7). It may also remit the need for antipsychotic polypharmacy in people with TRS.

Unfortunately, the prescribing of clozapine has been limited by several factors. Clozapine is a narrow therapeutic index drug and can cause potentially life-threatening adverse effects, including intestinal obstruction, myocarditis, and agranulocytosis, the latter of which has historically mandated strict neutrophil count monitoring (5, 8). Next, clozapine is predominantly metabolized by cytochrome P450 (CYP) CYP1A2, CYP3A4, and CYP2D6 to multiple metabolites, including an active metabolite norclozapine, and is subject to drug-drug interactions that can lead to psychiatric decompensation or adverse effects. For example, aryl hydrocarbons present in cigarette smoke induce CYP1A2 activity, and people who smoke regularly may experience as much as a 50% increase in clozapine levels following smoking cessation (9, 10). The ratio of clozapine to norclozapine plasma levels, also known as the metabolic ratio, can indicate the presence of genetic polymorphisms, environmental exposures, or medications that influence CYP1A2 activity. However, routine monitoring of the metabolic ratio to predict adverse effects or therapeutic response can be challenging as the ratio can shift unpredictably following clozapine dose changes (9). While monitoring plasma clozapine levels is more common and can aid clinicians in identifying toxicity or medication non-adherence, individual levels can also fluctuate depending on patient-specific pharmacokinetic parameters (e.g., metabolism, genetics). The therapeutic range for plasma clozapine levels is generally regarded as 350 to 600 ng/mL, as studies suggest that most patients will not obtain additional benefit at levels exceeding 600 ng/mL, even though some laboratories report 1,000 ng/mL as the upper limit (11).

The impact of drug-drug interactions on clozapine safety and efficacy is critical in the context of tuberculosis treatment. People with serious mental illness are at higher risk of acquiring tuberculosis compared to the general population, and comorbid mental illness has been associated with poorer tuberculosis recovery outcomes (12). The preferred anti-tuberculosis therapy (ATT) for drug-susceptible tuberculosis consists of 2 months of daily rifampicin, isoniazid, pyrazinamide, and ethambutol followed by 4 months of daily rifampicin and isoniazid (13). Daily observed therapy (DOT), in which a healthcare worker, either virtually or in person, watches a patient take their ATT and documents completed doses, can optimize adherence and should be offered for all people receiving ATT, especially those whose clinical course may be impacted by drug-drug interactions. Of note, rifampicin and isoniazid can both affect clozapine levels via their respective induction and inhibition of multiple CYP450 isoforms, including CYP1A2 and CYP3A4 (14). Further complicating clinical decision making is evidence that acute infection itself can increase clozapine levels and the risk of toxicity, particularly in the early phases of tuberculosis (9, 15).

There are few reports describing the co-management of clozapine and ATT and no formal treatment guidelines on this topic. Here, we review the existing literature, present a successful case of clozapine management in an adult with schizophrenia receiving rifampicin and isoniazid for new-onset tuberculosis, offer suggestions for managing clozapine in ATT, and propose changes to pertinent guidelines.

## Literature review

We identified 6 articles examining drug-drug interactions between clozapine and ATT. Five of these were case reports and are summarized below.

Gee et al. describe a case of a 28-year-old non-smoker with schizoaffective disorder who was taking clozapine 400 mg daily in March 2012 when they began an empiric course of rifampicin, isoniazid, pyrazinamide and moxifloxacin for presumed abdominal tuberculosis (16). Prior to starting ATT, the patient’s clozapine levels generally ranged from 240–360 ng/mL. Upon starting ATT, the clozapine dose was empirically increased by 25% to 500 mg daily. The clozapine levels became sub-therapeutic within 12 days of initiating rifampicin and continued to decline to 100 ng/mL. This decline was accompanied by worsening mutism and agitation. The clozapine dose was increased to 800 mg daily, or a near doubling from baseline, resulting in levels of approximately 650 ng/mL. However, the levels quickly declined after 3 weeks to approximately 150–350 ng/mL for the remainder of ATT, despite increasing the clozapine dose to 1000 mg daily. Upon discontinuing ATT, the clozapine dose was prospectively halved and tapered by 50 mg/day over 10 days. The clozapine levels ultimately returned to therapeutic range with resolution of psychiatric symptoms at a dose of 550 mg daily. The authors recommend measuring baseline clozapine levels and preemptively increasing the dose upon initiating rifampicin, monitoring clozapine levels at least once weekly during therapy, and tapering the clozapine dose over 7–10 days following discontinuation of rifampicin to prevent adverse events.

Peritogiannis et al. report a case of a 30-year-old with schizophrenia who was prescribed a 6-month course of rifampicin 600 mg daily for pulmonary tuberculosis and maintained on clozapine 300 mg daily (17). The patient’s smoking status was not documented. After two weeks, the patient experienced reduced sialorrhea and sedation but relapse of their psychiatric symptoms. The clozapine dose was titrated to 550 mg daily in the following month with minimal improvement in their psychiatric symptoms. Sedation, sialorrhea and clinical efficacy returned within 7 days of completing rifampicin and the clozapine dose was reduced to 500 mg daily. Clozapine levels were not measured due to laboratory technical difficulties. The authors endorse individualization of clozapine doses during ATT but do not issue specific dosing or monitoring recommendations.

Joos et al. report a case of a 33-year-old smoker with schizophrenia taking clozapine 400 mg daily at the time of starting rifampicin 600 mg daily, isoniazid 300 mg daily, pyrazinamide 2000 mg daily, and ethambutol 1600 mg daily for pulmonary tuberculosis (18). Three weeks after initiating ATT, their clozapine levels declined from a baseline level of 240 ng/mL to approximately 80 ng/mL. This decline was accompanied by restlessness and delusional thinking. Clozapine was increased by 50% to 600 mg daily with minimal improvement in psychiatric symptoms or clozapine levels. Rifampicin was switched to ciprofloxacin 1000 mg/day; within 3 days, the patient’s clozapine levels returned to therapeutic range with improvement of their psychiatric symptoms. The authors concluded that clozapine levels declined by 600% 2–3 weeks after the initiation of rifampicin due to CYP enzyme induction but increased following discontinuation of rifampicin due to CYP enzyme inhibition by isoniazid. The authors recommend monitoring clozapine levels upon discontinuation of rifampicin but do not provide titration or monitoring schedules.

Grover et al. describe a case of a 31-year-old non-smoker with schizophrenia who had been on clozapine 125 mg daily for 7 years with clinical stability (19). The patient was diagnosed with pulmonary tuberculosis and initiated on rifampicin 600 mg daily, isoniazid 300 mg daily, pyrazinamide 1400 mg daily, and ethambutol 1000 mg daily. After two months of ATT, the patient developed persecutory delusions and auditory hallucinations that progressed despite more than doubling the clozapine dose to 300 mg daily. Isoniazid was switched to levofloxacin 750 mg daily, which may have contributed to five months of persistent psychiatric symptoms and subsequent admission to an inpatient behavioral health unit. The patient’s symptoms began to improve 6 weeks following completion of ATT, and they were discharged on clozapine 300 mg daily. The authors do not report clozapine levels or issue monitoring or titration recommendations.

Angelini et al. describe a case of a 65-year-old with schizophrenia and unknown smoking status maintained on clozapine 200 mg twice daily who was initiated on a 9-month course of isoniazid 300 mg daily for tuberculosis (20). Their pre-treatment clozapine level was 397 ng/mL. Nine days after initiating isoniazid, the clozapine level increased by 90% to 756 ng/mL, and the clozapine dose was decreased by 25% to 150 mg twice daily. On day 20 of isoniazid therapy, the clozapine level declined to 527 ng/mL, and the dose was further reduced by 50% from baseline to 100 mg twice-daily. Fifty-four days after discontinuing isoniazid, the clozapine level decreased to 239 ng/mL, and the patient was maintained on clozapine 100 mg twice-daily. However, one month later, the patient exhibited worsening psychiatric symptoms, which resolved after returning to 200 mg twice-daily. The authors recommend monitoring clozapine levels but do not specify a dose titration or monitoring schedule.

## Case report

ZZ is a 35-year-old with a history of obesity, schizophrenia, psoriasis and tobacco use. ZZ was treated with clozapine for four years during which their dose was titrated to a maximum of 900 mg daily in response to ongoing, functionally impairing psychotic symptoms with no significant side effects from clozapine other than sialorrhea. At a routine outpatient psychiatry visit, their clozapine dose was decreased from 900 mg nightly to 800 mg nightly to address new concerns for weight gain; aripiprazole 5 mg daily was initiated to target persistent auditory hallucinations and delusions. At this visit, their clozapine and norclozapine levels were 972 and 358 ng/mL, respectively.

**Figure 1:**
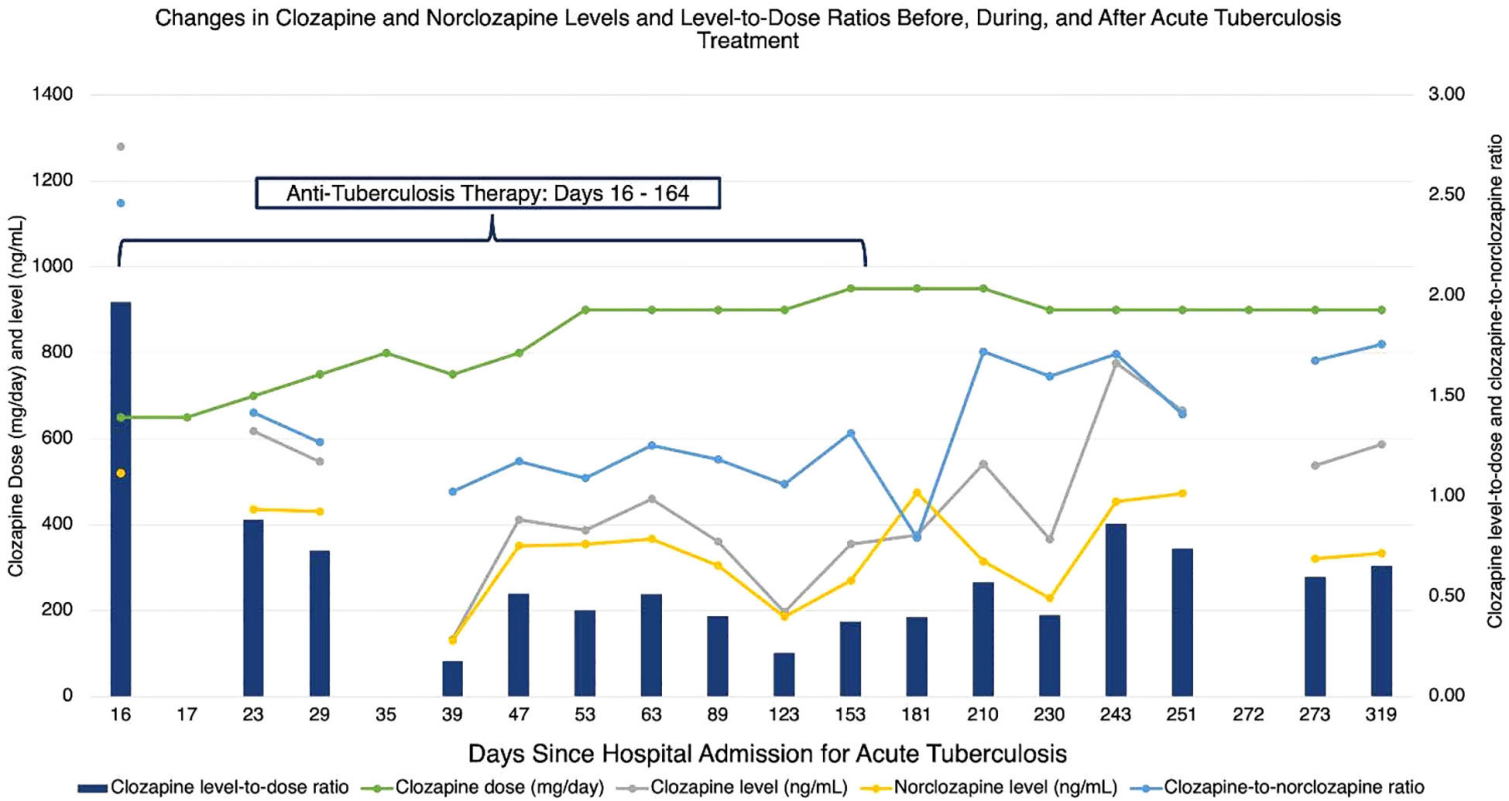
Changes in clozapine and norclozapine levels and level-to-dose ratios before, during, and after acute tuberculosis treatment.

ZZ was admitted to the hospital 18 days later for nausea, vomiting, cough, and malaise. Although ZZ denied adverse effects, on hospitalization day 6, their clozapine dose was decreased by nearly 20% from 800 mg nightly to 650 mg nightly to avoid clozapine toxicity in the setting of acute infection and cessation of smoking during hospitalization. After a period of diagnostic uncertainty, pulmonary tuberculosis was diagnosed by acid-fast bacilli stain and Mycobacterium tuberculosis positive culture from a sputum sample. On hospital day 16, ZZ began a 6-month course of ATT: 6 months of rifampicin and isoniazid; pyrazinamide and ethambutol stopped after 7 and 9 weeks respectively following negative sputum cultures and testing that confirmed sensitivity to rifampicin and isoniazid. In anticipation of CYP450 enzyme induction by rifampicin, ZZ’s clozapine dose was gradually increased by 25 mg daily to 750 mg nightly; aripiprazole was titrated to 15 mg daily over the following 3 weeks. These dose adjustments were made in collaboration with a clinical pharmacist. Clozapine and norclozapine trough levels were drawn at least once weekly and ZZ was regularly seen by the inpatient psychiatry consult team throughout the hospitalization. Although ZZ exhibited some worsening delusional content following the initial dose decrease, they did not deteriorate in their clinical status. On hospital day 35, ZZ was discharged on clozapine 750 mg nightly and aripiprazole 15 mg daily alongside daily observed ATT. ZZ has been seen by their outpatient psychiatrist and had clozapine and norclozapine levels drawn at least once monthly since discharge. Over the following 6 months, their clozapine dose was adjusted to 950 mg nightly and their aripiprazole to 20 mg daily for worsening intensity in delusional content. These adjustments were also made in consultation with a clinical pharmacist. Following these dose increases, ZZ reported drooling which was managed with the addition of glycopyrrolate but maintained stability in their psychiatric symptoms. ATT was completed after 6 months. ZZ’s clozapine and norclozapine levels increased appreciably 13 days afterwards and the clozapine dose was adjusted downwards by 50 mg per day 19 days following discontinuation of ATT (See **Figure 1**). ZZ’s psychiatric and ATT doses are listed in **Table 1**, while their absolute neutrophil counts and clozapine and norclozapine levels are listed in **Table 2**.

**Table 1:** Psychiatric and anti-tuberculosis medication doses.

Time	Medication (mg/day)					
	Clozapine	Aripiprazole	Rifampicin	Isoniazid	Pyrazinamide	Ethambutol
Days Before Hospitalization						
17	800	5				
Day of Hospitalization						
6	650	10				
15	650	10	600	300	1500	1200
19	700	10	600	300	1500	1200
20	750	10	600	300	1500	1200
30	750	15	600	300	1500	1200
Days After Hospitalization						
1	800	15	600	300	2000*	1600*
2	750	15	600	300	2000	1600
5	800	15	600	300	2000	1600
15	900	15	600	300	2000	1600
19	900	20	600	300	2000	1600
29	900	20	600	300	2000	**Discontinued**
47	900	20	600	300	**Discontinued**	**--**
93	950	20	600	300	--	--
164	950	20	**Discontinued**		**--**	**--**
180	900	20	--	--	--	--
Years After Hospitalization						
1.5 years	900	20	--	--	--	--

*The doses of pyrazinamide and ethambutol were increased in accordance with weight-based dosing recommendations.

Gray color indicates they were not receiving that treatment at that time.

The bold values are to indicated when certain treatments were discontinued.

**Table 2:** Clozapine doses, levels and ratios before, during, and after anti-tuberculosis treatment.

Days since hospital admission	Days since initiating anti-TB treatment	ANC (cells/µL)	Clozapine (ng/mL)^1^	Norclozapine (ng/mL)	Clozapine-to-Norclozapine ratio^2^	Clozapine dose (mg/day)	Clozapine level-to-dose ratio
-58	-72		972	358	2.71	900	1.08
16	2		1280	520	2.46	650	1.97
23	9	2720	618	436	1.42	700	0.88
29	15		547	431	1.27	750	0.73
35	21					800	
39	25	4090	134	131	1.02	750	0.18
47	33	2550	412	351	1.17	800	0.52
53	39	2520	387	355	1.09	900	0.43
63	49	2030	460	367	1.25	900	0.51
89	75	2410	361	305	1.18	900	0.40
123	109	2320	197	186	1.06	900	0.22
153	139	1830	355	270	1.31	950	0.37
181	167	2140	376	475	0.79	950	0.40
210	196	2150	542	315	1.72	950	0.57
230	216	2640	366	229	1.60	900	0.41
243	229		776	454	1.71	900	0.86
251	237	2280	666	473	1.41	900	0.74
273	259	2210	538	321	1.68	900	0.60
319	305	2140	587	334	1.76	900	0.65

^1^In clinical practice, 350 ng/mL is considered the lower threshold for clozapine efficacy. While some laboratories designate 600 ng/mL as the upper threshold, patients may experience clinical benefit at levels as high as 1000 ng/mL.

^2^A clozapine-to-norclozapine ratio below 1.32 may indicate CYP1A2 induction by medications (such as carbamazepine) or smoking. A ratio above 2.00 may indicate CYP1A2 inhibition by medications (such as fluvoxamine) or acute changes in clozapine metabolism during medical illness.

Gray color means the lab value was not available or not drawn/collected.

In reflecting on their experience, ZZ appreciated that their wishes were taken into account during their diagnostic workup and treatment. Specifically, they recalled being grateful that their wishes around avoiding a mediastinoscopy during initial diagnostic investigation were honored. While they understood and were agreeable to their treatment plan, they also shared that they were frustrated by their lengthy hospital stay; this inpatient stay, more than subsequent daily outpatient ATT, was particularly frustrating for ZZ. ZZ was hopeful that their experience, and this article, could be helpful to others in a similar situation.

## Discussion

While there are few case reports describing the co-management of clozapine and ATT, our case report and the series summarized above illustrate common themes: patients who were previously stable on clozapine are at risk of worsening psychiatric symptoms, alongside declining clozapine levels, after starting rifampicin-containing ATT. Specifically, clozapine levels decline 1–3 weeks after starting rifampicin, often necessitating clozapine dose increases by as much as 50-150%, and recover only gradually, often more than 30 days, after rifampicin cessation. In the absence of comprehensive studies in this area, we offer the following recommendations:

We recommend monitoring clozapine levels at least once weekly during rifampicin therapy and for 8–12 weeks following its cessation, particularly for patients who are exposed to environmental factors that affect clozapine disposition (e.g., cigarette smoking or medications that influence CYP1A2 activity). Ideally, clozapine levels can be timed synergistically with neutrophil monitoring or DOT visits to minimize laboratory burden. However, we acknowledge that socioeconomic factors, including housing insecurity and geographic inaccessibility to an outpatient behavioral health provider, can influence the feasibility of this monitoring and titration schedule. In these circumstances, stability in clozapine levels and the patient’s symptoms during the first 4 weeks of therapy may allow for once monthly monitoring. The remittance of adverse effects, such as sialorrhea, could indicate a reduction in clozapine levels and prompt recurrent monitoring. We favor adjusting clozapine in larger than typical increments of 50–100 mg/day given the significance of CYP450 enzyme induction by rifampicin. While preemptive dose increases in clozapine could be necessary in some circumstances such as limited follow up or slow lab turnaround times, preferably clozapine doses should be adjusted based on close monitoring of clozapine levels. This is especially true early in treatment when the level is difficult to predict due to complex patient-specific factors (e.g. inflammatory effects, the specific ATT regimen, initial clozapine dose). If a patient’s clozapine dose requirement during ATT exceeds or is anticipated to exceed 900 mg daily, the addition of a second antipsychotic that is primarily metabolized by CYP2D6 (e.g., aripiprazole) or one with minimal CYP metabolism (e.g., amisulpride) could be considered. We recommend that psychiatric practitioners consult with an infectious disease specialist and a clinical pharmacist to weigh the risks and benefits of rifamycin-sparing regimens, which could interact less significantly with clozapine. Finally, we encourage the inclusion of the clozapine-ATT interaction in tuberculosis treatment and general antimicrobial reference guides.

The findings observed in our patient case are explained largely by rifampicin’s impact upon clozapine metabolism, specifically delayed CYP450 enzyme induction following rifampicin initiation and delayed CYP450 enzyme de-induction upon its cessation. CYP1A2 and CYP3A4 are major substrate pathways, accounting for approximately 30 – 55% and 20% of clozapine biotransformation, respectively. CYP2C19 and CYP2D6 are also involved as minor substrate pathways (9). Rifampicin is a moderate inducer of multiple CYP450 enzymes but is an especially potent inducer of CYP3A4 (21, 22). Enzyme induction due to rifampicin can be significant and unpredictable enough to require substantial dose modifications of concomitant CYP450 substrates or avoidance of rifampicin altogether. For instance, rifampicin is contraindicated in people taking most protease inhibitors due to the risk of antiviral failure (23). Rifampicin’s inductive effects may also be potent enough to outweigh CYP450 inhibition by other medications. Although isoniazid is a moderate inhibitor of CYP2C9 and CYP3A4, our literature review suggests that co-administration of isoniazid and rifampicin results in net enzyme induction and enhanced metabolism of CYP450 substrates such as clozapine.

Managing the interaction between clozapine and ATT can be challenging for several reasons. Firstly, beyond the small number of published case reports, there is limited evidence to inform clozapine titrations or monitoring during ATT; the 2024 tuberculosis guidelines published by the American Thoracic Society, Centers for Disease Control and Prevention, European Respiratory Society, and Infectious Diseases Society of America (ATS/CDC/ERSIDSA), for instance, make no mention of clozapine (13). Secondly, rifamycin-based regimens are among the most effective and shortest treatments for drug-susceptible tuberculosis. Thirdly, as clozapine is typically reserved for patients who have not responded to multiple antipsychotics, switching clozapine to circumvent drug-drug interactions is not pragmatic for most patients. Adding a second antipsychotic during antituberculosis therapy may protect against decompensation. However, options are limited as most antipsychotics are at least partially metabolized by the CYP450 enzyme system (9, 24). Next, a range of additional factors, including genetic polymorphisms, pregnancy, smoking status, and acute infection itself, can influence CYP450 enzyme activity (9, 25). Finally, people with serious mental illness are at higher risk of being lost to follow-up, experiencing poorer tuberculosis recovery outcomes, and inhabiting environments associated with enhanced tuberculosis transmission (such as correctional facilities and housing shelters) than the general population. Delaying treatment in this population can have grave consequences for both the individual patient and public health (26).

Our case adds to the body of literature and provides important insights into the co-management of clozapine and ATT. ZZ’s clozapine levels increased between hospitalization and ATT initiation. We attribute this to smoking cessation during hospitalization and the impact of clozapine on inflammatory cytokines and CYP450 enzyme system. Severe infection and clozapine itself can lead to alterations in interleukin-6, interleukin-1, interferon-ɣ and tumor necrosis factor-α leading to decreases in CYP1A2 and the body’s capacity to metabolize clozapine (8, 14, 24, 27). Additionally, clozapine’s effects on muscarinic and histaminic receptors and cytokine signaling cascades may cause immunosuppressant effects that, in turn, render patients more vulnerable to acquiring tuberculosis. Clozapine’s immunologic impacts may explain observations that clozapine led to an increased risk for tuberculosis and can lead to reactivation of latent tuberculosis (28).

As anticipated, ZZ’s clozapine levels steadily declined 9 days after starting ATT. The clozapine levels and clozapine-to-norclozapine ratios remained below pre-treatment levels for the remainder of ATT, reflective of ongoing CYP450 enzyme induction. On days 53 and 123 of ATT, the treating psychiatrist noted an increase in delusional thought content and increased the clozapine dose by 50 mg daily. This mild worsening of psychiatric symptoms may be explained by declines in the clozapine-to-norclozapine ratios. Multiple factors, including possible resumption of smoking or changes in inflammatory cytokine signaling during ATT, could explain this decline, While the investigators in prior case reports overcame lower clozapine levels or ratios by doubling the clozapine dose, ZZ’s pre-treatment clozapine dose was near the typical dose ceiling of 900 mg per day, rendering a dose increase of this magnitude infeasible and potentially unsafe. In contrast to prior case reports, ZZ’s clozapine dosages were only adjusted upwards by 15-20% from baseline. Proactive increases in ZZ’s aripiprazole dosage may have protected against psychiatric decompensation, as aripiprazole is predominantly metabolized by CYP2D6. Our case suggests that adjunctive antipsychotic therapy may be effective when baseline clozapine doses are near the recommended daily maximum. Of note, aripiprazole prescribing recommendations recognize metabolism by CYP3A4 and suggest the dose be increased in the presence of strong CYP3A4 inducers (29).

ZZ’s course following the completion of ATT also differs from prior case reports. While ZZ’s clozapine levels and clozapine-to-norclozapine ratio increased after rifampicin therapy ended, they did not reach pre-treatment levels, despite ZZ’s clozapine dose being higher than pre-treatment. In contrast to prior reports, ZZ did not require dramatic clozapine dose adjustments following discontinuation of rifampicin to prevent clozapine toxicity. ZZ also received most of their ATT in the outpatient setting, via directly observed therapy, to ensure adherence. Our case highlights the need for close collaboration among multiple disciplines across transitions of care, both inpatient and outpatient, including consult/liaison and outpatient psychiatry services, local or territorial public health services, infectious disease specialists, and pharmacy services. ZZ continues to exhibit psychiatric stability and adherence to clozapine and aripiprazole.

### Limitations

While our case report has key advantages, including the reporting of medication doses and clozapine and norclozapine levels before, during and after ATT, there are limitations. The trends observed in ZZ may be partially explained by non-medication factors, including genetics and smoking status, and therefore may not be applicable to all patients receiving clozapine. Similarly, while the combination of clozapine and aripiprazole appears to have been effective for our patient, we cannot definitively conclude that antipsychotic polypharmacy will protect against psychiatric decompensation in people receiving clozapine and standard ATT.

Finally, while we offer recommendations, we emphasize these are based on the case reports and literature reviewed here and represent the opinions of the authors. Additional research, as well as soliciting recommendations from larger groups of clinicians and experts would strengthen and refine the recommendations proposed.

## Conclusion

Our case and literature review underscore important issues in the management of ATT among adults receiving clozapine. Clozapine levels can fluctuate dramatically in response to acute illness, smoking status, and the initiation of rifamycins, but these shifts may not always be accompanied by adverse effects or worsening psychiatric symptoms. Additionally, these fluctuations may be delayed by as much as 3 weeks following rifampicin initiation; clinicians should be mindful of levels slowly returning to concentrations seen prior to ATT after rifampicin is discontinued. When feasible, routine monitoring of clozapine and norclozapine levels can be useful to capture the magnitude of the drug interaction and guide clozapine dosing during and after ATT. Close monitoring of a patient’s psychiatric status is necessary as recurrence of symptoms is not uncommon after starting ATT. In our experience, pre-emptive dose adjustments, frequent monitoring of levels, and close outpatient clinical assessments were all necessary to maintain clinical stability and prevent clozapine toxicity.

## Data Availability

The original contributions presented in the study are included in the article/supplementary material. Further inquiries can be directed to the corresponding authors.
